# Sub-2 Å cryo-EM structures of transcribing RNA polymerase II reveal critical roles of water molecules in catalysis

**DOI:** 10.1016/j.molcel.2026.04.007

**Published:** 2026-04-30

**Authors:** Qingrong Li, Gangshun Yi, Yue Wu, Sophy Xu, Jenny Chong, Xuhui Huang, Peijun Zhang, Dong Wang

**Affiliations:** 1 Department of Pharmaceutical Sciences, Skaggs School of Pharmacy and Pharmaceutical Sciences, University of California, San Diego, La Jolla, CA 92093, USA; 2 Division of Structural Biology, Centre for Human Genetics, University of Oxford, Oxford OX3 7BN, UK; 3 Diamond Light Source, Harwell Science and Innovation Campus, Didcot OX11 0DE, UK; 4 Department of Chemistry, University of Wisconsin-Madison, Madison, WI 53706, USA; 5 Chinese Academy of Medical Sciences Oxford Institute, University of Oxford, Oxford OX3 7BN, UK; 6 Department of Molecular and Cellular Medicine, School of Medicine, University of California, San Diego, La Jolla, CA 92093, USA; 7 Department of Chemistry and Biochemistry, University of California, San Diego, La Jolla, CA 92093, USA

## Abstract

RNA polymerase II (RNA Pol II) is central to gene expression, but its catalytic mechanism remains elusive due to the absence of high-resolution structural data. The role of water molecules in RNA Pol II catalysis is unknown. Here, we present 3 high-resolution cryo-electron microscopy structures of active *Saccharomyces cerevisiae* RNA Pol II elongation complexes in distinct catalytic states: two pre-catalysis states at 1.96 Å and 2.26 A resolution and a post-catalysis state at 2.33 Å resolution. Each structure contains over 700–1,350 ordered water molecules, many located at functionally critical positions. Comparative analysis shows that these waters play essential roles in proton-transfer steps during RNA Pol II catalysis, facilitating substrate recognition and trigger-loop folding during nucleotide addition. Strikingly, these waters are conserved between prokaryotic and eukaryotic transcription machineries (see Mueller and Darst). These findings provide unprecedented mechanistic insights into RNA Pol II catalysis and reveal vital and evolutionarily conserved roles of water molecules in transcription.

## Graphical abstract

**Graphical abstract:**
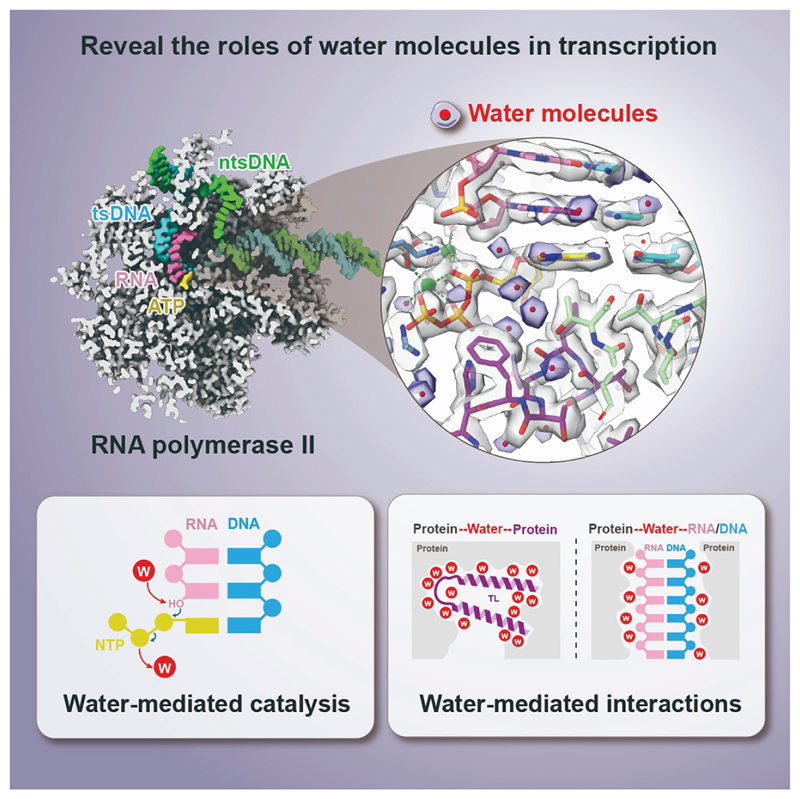

## Introduction

RNA polymerase II (RNA Pol II) is a central transcription machinery in the first step of gene expression, and it synthesizes pre-mRNA, non-coding RNAs, and small nucleolar RNAs (snoR-NAs).^1–3^ However, our current understanding of RNA Pol II transcription catalytic mechanism remains incomplete. Previous research has revealed that a mobile, conserved motif, termed trigger loop (TL), folds into a closed state upon cognate substrate binding.^3–8^ This pre-catalysis state (substrate-bound, TL-closed state) is essential for aligning active site residues, RNA primer, substrate, and metal ions in a reaction-ready configuration.^5,9–11^ However, despite extensive research efforts over the past 25 years, only 2 pre-catalysis state crystal structures of the RNA Pol II elongation complex (EC) were determined at resolutions lower than 3.0 Å, largely due to the mobile nature of TL as well as technical limitations in X-ray and cryo-electron microscopy (cryo-EM) approaches.^5,12^ Due to limited resolution and crystal packing effects, these structures left uncertainty on the configurations of active site, as well as on the correct substrate and metal coordination poised for reaction, and failed to resolve the complete transcription bubble.^5,12^ The functional roles of key residues for RNA Pol II transcription remain ambiguous.

It is proposed that the nucleotide addition reaction occurs via an S_N_2 mechanism.^13^ However, the molecular mechanism of proton transfer steps, the center piece of catalytic mechanism of RNA Pol II transcription, remains uncertain. The identities of the proton acceptor for deprotonation of 3′-OH group of the RNA primer (before nucleophilic attack) and the proton donor for pyrophosphate (PPi) product (after nucleophilic attack) remain unclear.

Finally, our current understanding of RNA Pol II transcription is protein-centered. While emerging evidence suggests that water acts as an essential and active participant in maintaining structure, stability, dynamics, and function of biomolecules,^14–19^ the functions of water molecules in transcription machinery remain largely unexplored and underappreciated as the “dark matter” of transcription, mainly due to lack of high-resolution structures of RNA Pol II ECs. In particular, the roles of water molecules in RNA Pol II catalysis, substrate recognition, and protein-nucleic acids interactions are poorly understood.

A direct visualization of water densities requires a resolution better than 2.5 Å. To overcome the previous challenges in obtaining high-resolution cryo-EM structure of RNA Pol II ECs, in particular preferred orientation and air-water interface denaturation,^20^ we developed a cryo-EM affinity grid with monodispersed single-particle streptavidin (mspSA) on lipid monolayers and a tethering method to attach RNA Pol II ECs to mspSA grids.^21^ Using streptavidin-affinity grids and optimized data collection strategies, we have successfully determined 3 high-resolution cryo-EM structures of RNA Pol II EC, 2 in pre-catalysis (at 1.96 and 2.26 Å resolution, respectively) and 1 post-catalysis states (at 2.33 Å resolution). These structures reveal a complete and detailed map of a fully assembled active site and over 700–1,350 well-resolved ordered water molecules. Our work unveils unprecedented and critical roles of water molecules in substrate recognition and the catalytic mechanism of transcription. Strikingly, these functional waters are highly conserved between prokaryotic and eukaryotic multi-subunit RNA polymerases (see Mueller and Darst^22^). The elucidation of a complete active site of transcription machinery reveals that functional waters are evolutionarily conserved and integral components of transcription machinery with critical roles in transcription catalysis, which marks a major conceptual leap beyond the traditional “protein-centered” paradigm of transcription.

## Results

### Cryo-EM RNA Pol II EC structures at pre-catalysis state unambiguously define the reaction-ready configurations of a complete active site

**Figure 1:**
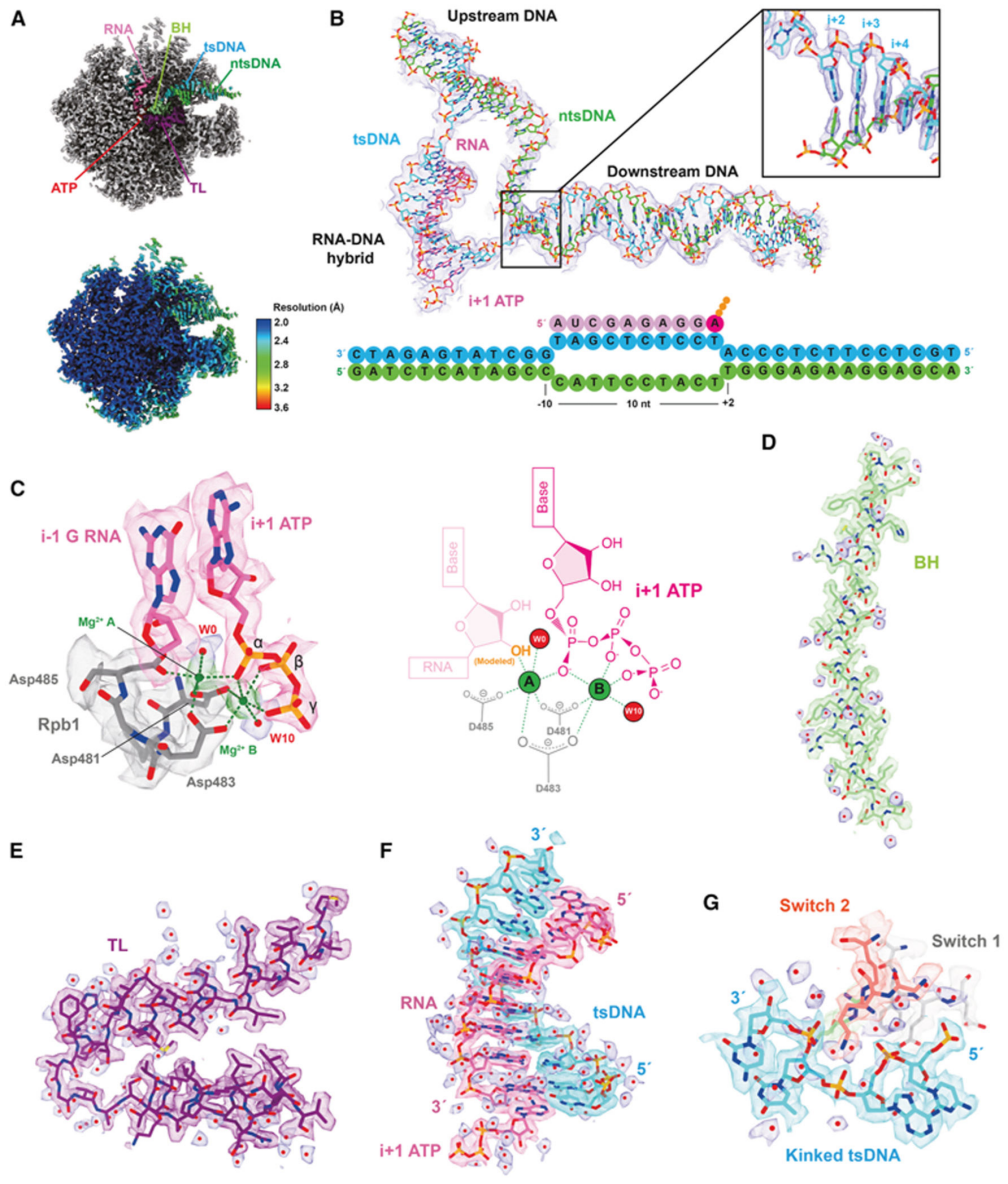
High-resolution cryo-EM structure of RNA Pol II EC at the pre-catalysis state with substrate ATP binding (A) RNA Pol II elongation complex (EC) structure at 1.96 Å resolution. Color code and abbreviation: RNA (hot pink), template DNA (tsDNA cyan), non-template DNA (ntsDNA, lime), bridge helix (BH, light green), trigger loop (TL, purple), and ATP (red). Bottom: local resolution map from 2.0 Å (blue, high) to 3.6 Å (red, low). (B) Cryo-EM density and scheme of the complete transcription bubble in the RNA Pol II EC. (C–G) Cryo-EM densities of selected regions, including (C) the active site (gray) showing two Mg^2+^ ions (green) and ATP substrate (hot pink), (D) BH, (E) TL, (F) RNA-DNA hybrid, and (G) kinked tsDNA interacting with switch loop 1 (silver) and switch loop 2 (salmon). Cryo-EM density maps are contoured at 5-σ (contour level = 0.12). In (C), the 3′-OH group (not present in the structure) was modeled onto the RNA primer to illustrate the expected coordination with metal A.

We determined a cryo-EM structure of a cognate substrate-bound RNA Pol II EC at the pre-catalysis state with a global resolution of 2.26 Å(Figure S1; Table 1). We further improved the resolution of RNA Pol II EC at the pre-catalysis state to 1.96 Å by including the elongation factor Elf1 (with the highest local resolution reaching 1.92 Å near the active site) (Figure 1A; Figure S2; Table 1; Video S1). Both pre-catalysis RNA Pol II EC structures are captured in a substrate-bound, TL-closed state (highly consistent with 0.34 Å root-mean-square deviation [RMSD] at the active site) and reveal a fully ordered complete transcription bubble with clear upstream and downstream bubble edges (Figure 1B; Figure S3; Video S1). These structures—including a bound substrate at the addition site (Figure 1C; Video S1), a bent bridge helix (BH) (Figure 1D; Video S1), a fully closed TL (Figure 1E; Video S1), the RNA-DNA hybrid (Figure 1F; Video S1), and the kinked template DNA (tsDNA) (Figure 1G; Video S1), with all side chains and ordered water molecules unambiguously resolved— define the pre-catalysis active site.

**Figure 2:**
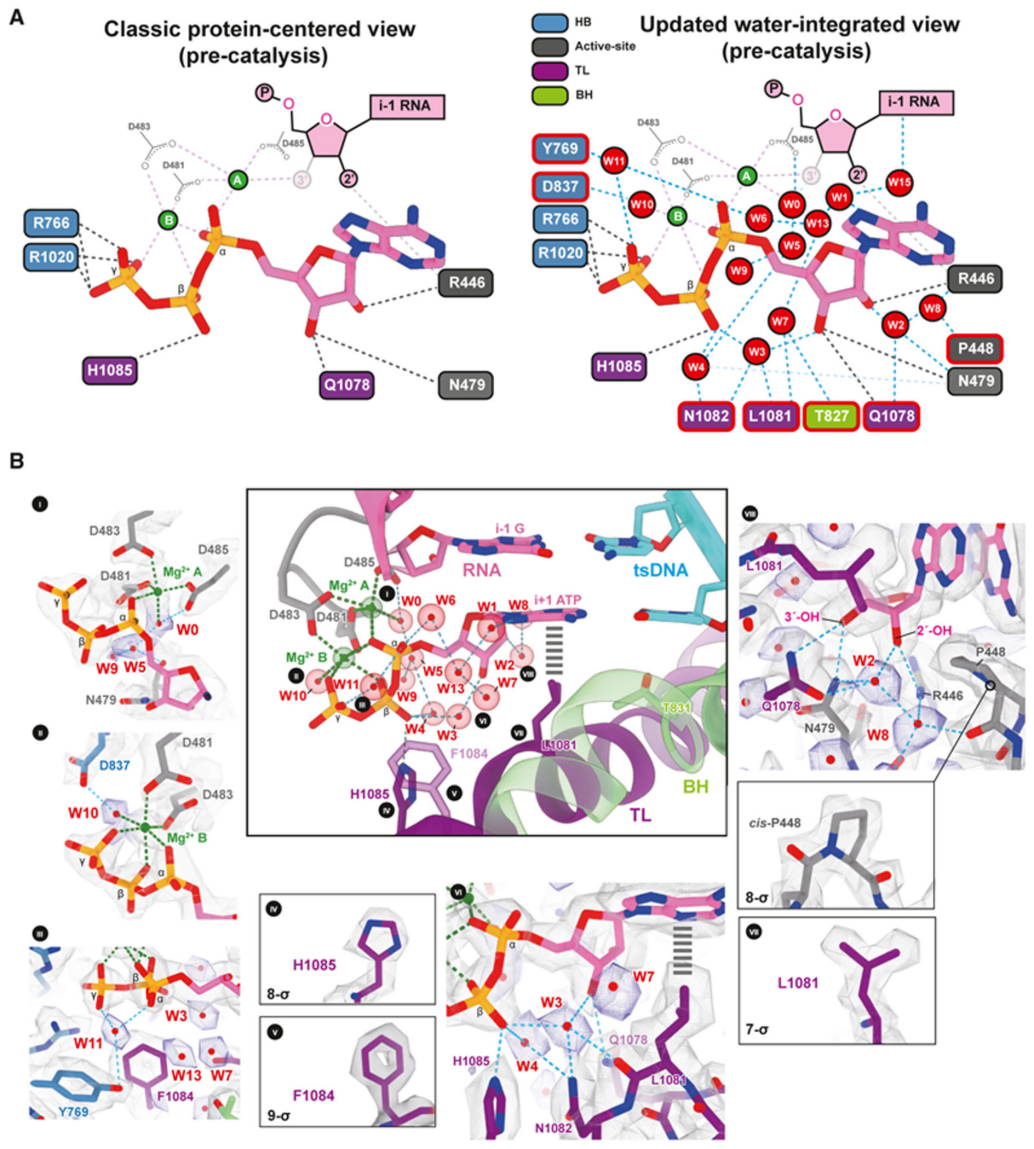
Water molecules mediate interactions between substrate ATP and the catalytic center at the pre-catalysis state (A) Hydrogen-bonding interactions between the substrate ATP (hot pink) and the catalytic center in the classic protein-centered view (left) and updated water-integrated view (right). Residues from different domains are color-coded as indicated. Water molecules are highlighted as red spheres. The residues that interact with ATP via water molecules are outlined with red borders (identified in this study). Water-mediated hydrogen bonds are shown as blue dashed lines; direct protein-substrate hydrogen bonds as gray dashed lines; and Mg^2+^-mediated coordination bonds as light purple dashed lines. A semi-transparent 3′-OH group was modeled onto the RNA primer to illustrate the expected coordination with metal A. (B) Cryo-EM structures of water-mediated interactions within the RNA Pol II substrate recognition network. The middle panel displays the spatial distribution of water molecules (transparent red spheres) involved in substrate recognition. Hydrogen bonds and coordination bonds are indicated by blue and green dashed lines, respectively. Insets (I–VIII) present close-up views of local regions with cryo-EM density maps (shown as mesh). Contour levels are indicated in panels highlighting active-site side chains; all other close-up views are contoured at 5-σ. Thick dashed lines represent hydrophobic interactions between TL residue Leu1081 and the nucleobase of the substrate ATP in the middle panel and inset VI.

These high-resolution pre-catalysis RNA Pol II EC structures also define the reaction-ready configuration of a complete active site. The 1.96 Å cryo-EM map reveals an unambiguous density of Adenosine triphosphate (ATP) substrate with a chair-like triphosphate conformation that is precisely coordinated with two Mg^2+^ ions (Figure 1C; Video S1). Importantly, the octahedral coordination of metal B (Mg^2+^) is clearly resolved with tridentate coordination with all 3 phosphate groups (α, β, and γ phosphate) of the ATP substrate, which is different from previously reported low-resolution structures^5,12,23,24^ (Figure S4). A water molecule, W10, is found to coordinate with metal B as the sixth coordinate (Figures 1C and 2BII). The carboxyl groups of Asp481 (Rpb1) and Asp483 (Rpb1) bridge 2 Mg^2+^ ions (Mg^2+^ A and B), which also align closely with their counterparts in the DNA polymerase^25–27^ (Figures S4K–S4M). In addition to Asp481 and 483 residues, Mg^2+^ A is further coordinated with α phosphate of ATP substrate, Asp485, an ordered water molecule (W0), and presumably 3′-OH of RNA primer (Figures 1C and 2BI).

These high-resolution structures enable the determination of the rotamers and conformations of all key residue side chains and provide an accurate model for understanding RNA Pol II transcription (Video S1). The TL is in a fully closed conformation with 6 turns of the proximal half (from Met1063 to Asn1082) and 4 turns of the distal half (from Lys1092 to Val1107), forming a fully folded alpha helix (Video S1). We also clearly discerned the density of side chains and main chains of TL tip (from Thr1083 to Ser1091), which was poorly resolved in previous reported structures^5,12^ (Figure S5; Video S1; Table S1). The cryo-EM map clearly resolves the rotamers of all critical TL residues, including Gln1078, Leu1081, Asn1082, Phe1084, and His1085 (Figures 2A and 2BIV–2BVII; Figure S5). Key interactions and functional roles of these critical TL residues in the substrate recognition network are clarified (Figures 2A and 2B; Video S1). In addition, hydrophobic interactions at the TL tip—such as Phe1086(TL)-Val1089(TL)-Ile756(funnel) and Phe1084(TL)-Pro765 (hybrid binding domain, HB)-Tyr769(HB)-Leu842(BH)—are now visualized (Figures S5G and S5H). We further reveal that the minor-groove steric gate residue Pro448 adopts a *cis*-conformation (Figure 2BVIII, inset), in contrast to the ambiguous conformations reported in earlier RNA Pol II structures.

### Cryo-EM RNA Pol II EC structure unveils extensive water-mediated network of interactions between substrate and RNA Pol II catalytic center

Most importantly, all water molecules come into view in the 1.96 Å RNA Pol II EC structure, unveiling a previously unrecognized pivotal role of water molecules in mediating the substrate recognition network in RNA Pol II (Figure 2A). We identified 1,357 ordered waters (with a Q score higher than 0.7 and a density value above 4-σ) and 1 monovalent metal ion located at protein-protein and protein-nucleic acids interfaces of the RNA Pol II EC (Figure S6; Data S1 and S2). Among these waters, 13 are at the active site, whose mechanistic roles are described below (Figure 2).

In contrast to the traditional protein-centered view (Figure 2A, left), we now report that 13 ordered water molecules form interaction networks that connect all moieties of the substrate to key active-site residues (Figure 2A, right). The involvement of these water molecules greatly expands the current understanding of the substrate interaction network by incorporating 7 additional residues from distinct domains: Gln1078, Leu1081, and Asn1082 from the TL, Pro448 from the active-site domain, Tyr769 and Asp837 from hybrid binding (HB) domain, and

Thr827 from the bridge helix (BH) (Figure 2B, residues with red rectangle outline). Notably, these residues have previously been examined by biochemical and mutagenesis studies, yet the structural basis for their functional effects remains unclear.

**Figure 3:**
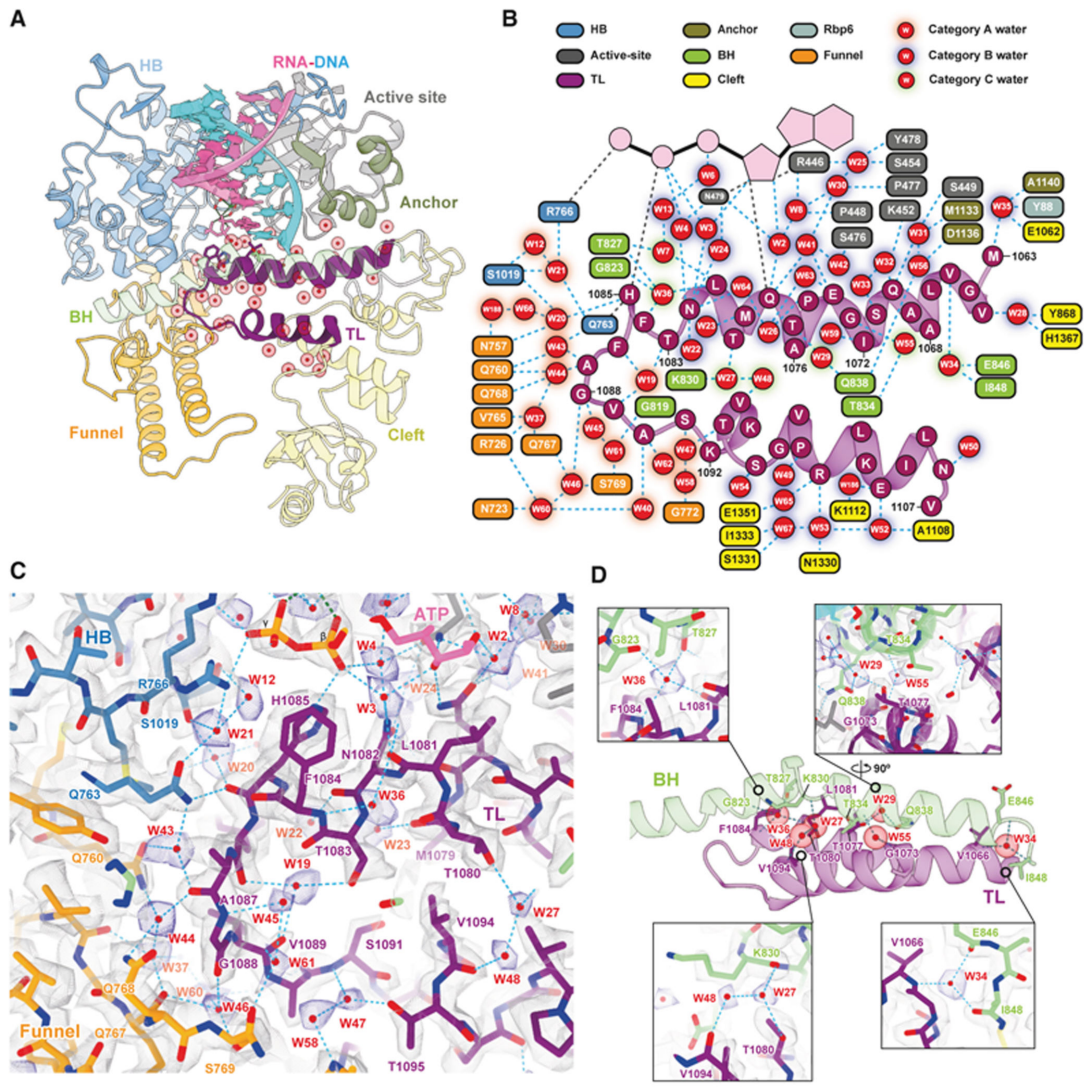
Water molecules engage in TL folding (A) Ordered waters (red spheres) surround the folded TL at the RNA Pol II active site. The TL tip inserts into a pocket between the funnel (orange) and hybrid-binding (HB, steel blue) domains. (B) Water-mediated hydrogen-bonding network surround the folded TL at the RNA Pol II active site. Water molecules are classified into 3 categories: category A (red sphere with orange rim), category B (red sphere with blue rim), and category C (red sphere with green rim). The representations of hydrogen bonds and coordination bonds are consistent with those in Figure 2A. (C) Water-mediated interactions that stabilize the TL tip and its contacts with funnel (orange, Rpb1) and HB (steel blue, Rpb2) domains. (D) Water-mediated interactions underlying the helical bundle formation between the TL and the BH. The middle panel shows the spatial distribution of water molecules involved in bundle formation, with 4 close-up views. All cryo-EM density maps in (C) and (D) are shown as mesh contoured at 5-σ.

Our water-integrated model provides a framework to rationalize these observations, for example, for mutations such as N479S,^5^ Q1078S,^28^ or N1082S,^28^ through disruption of water-mediated interactions (Figure S7; Table S2). Intriguingly, water-mediated interactions are important for RNA Pol II recognition of the sugar moiety of substrate (Figures 2A, 2BVI, and 2BVIII). In addition to previously reported direct interaction between the 2′-hydroxyl group (2′-OH) of ATP and Arg446,^5,6^ water-mediated interactions are between an ordered water molecule (W2) and the 2′-OH group of ATP and the amide groups of Asn479 and Gln1078 of the TL (Figure 2BVIII), supporting the findings from an early single-molecule fluorescence spectroscopy study that TL folding is a checkpoint for NTP ribo/deoxyribo discrimination.^29^ W2 further interacts with a water chain consisting of W8, W30, and W25, which in turn interacts with Arg446, Pro448, Pro477, Ser454, and Tyr478 on the active-site domain (Figures 3B and S5I). The 3′-OH of the substrate ATP is directly recognized by Gln1078 and Asn479 (Figures 2BVI and 2BVIII), clarifying previous ambiguity of these interactions.^12^ In addition, we identified a water molecule (W3) that acts as a hub in coordinating the 3′-OH and the *β*-phosphate group of ATP, the amide group of Asn1082, and the main chain of Leu1081 (Figure 2BVI).

In addition to sugar recognition, we also observed several waters interacting with the triphosphate moiety of ATP. The water molecule W6 bridges the *α*-phosphate of ATP to its nucleobase (N^7^) through a water network (W6-W13-W1), and additionally via W6-W13-W7 to TL residue Leu1081 and BH residue Thr827 (Figure 2A). For *β*-phosphate-group recognition, beyond the previously reported direct interaction with conserved His1085,^5^ here, we identified two water molecules, W3 and W4, that connect to the *β*-phosphate group of ATP, which can act as proton donors for proton transfer during nucleotide addition (see discussion section on RNA Pol II catalysis). These two water molecules mediate the interaction between the *β*-phosphate group and the TL with residues Leu1081 and Asn1082 (Figure 2BVI). In addition to the direct interactions formed by HB residues Arg766 and Arg1020 with the *γ* phosphate group, a water molecule (W11) is observed bridging the *α-* and *γ*-phosphate groups and mediating interactions with HB residue Tyr769 (Figure 2BIII).

Water molecules are important for Mg^2+^ ion coordination. Specifically, W0 coordinates with metal A and interacts with active-site residue Asp485, as well as connecting to a water chain that includes W5 and W9 (Figure 2BI). Notably, W0 may also serve as a proton acceptor to facilitate deprotonation of the 3′-OH of the RNA primer during nucleotide addition (see discussion on RNA Pol II catalysis). Similarly, water molecule W10 coordinates with metal B, completing its octahedral coordination. To our knowledge, a fully coordinated metal B in RNA Pol II has not been clearly observed in previous structures (Figure 2BII).

To evaluate the kinetic stability of resolved water molecules in the cryo-EM structure, we performed all-atom molecular dynamics (MD) simulations with position restraints applied to RNA Pol II heavy atoms, using the “residence time” of hydration sites as a straightforward metric. Key hydration sites centered on critical water molecules exhibited residence times that are significantly longer than bulk water (Figure S7). Most water molecules that mediate interactions between the ATP substrate and the RNA Pol II catalytic center (Figure 2A) demonstrated residence times ranging from 10^2^ to 10^4^ ps (for comparison, bulk water: 3 ps). These results highlight the critical role of water molecules in stabilizing substrate interactions at the active site.

### Water molecules are critical for TL folding and interactions with other catalytic center motifs

The high-resolution RNA Pol II EC structure also reveals that water molecules play a crucial role in stabilizing the closed TL conformation as well as its interactions with other key structural motifs, such as the BH, funnel, HB domain, anchor, cleft, and active-site domain (Figures 3A and 3B; Figure S5). We identified more than 50 water molecules that form extensive water-mediated hydrogen bonds that bridge residues of TL and other functional motifs in the active center (Figure 3B). These water-mediated interactions profoundly alter the canonical protein-centered view of TL function.

We divided these waters into 3 categories based on their function and location: category A water (total 17 water molecules; orange-red shade) that mediated interactions between TL tip (Thr1083-Ser1091) and funnel and HB domains; category B water (total 31 water molecules; blue shade) that mediated interactions between 2 stable stem helices of TL (Met1063-Asn1082 and Lys1092-Val1107) with substrate and other active center motifs; category C water (7 water molecules; green shade) that mediated interactions among TL and BH helices bundle (Figure 3B).

The category A water molecules are crucial for stabilizing the sharp-turn conformation of the TL in its fully closed state (Figure 3C; Figure S5). The TL tip region is flexible and can switch between a fully folded state and disordered open states during nucleotide addition. Upon TL folding, the TL tip inserts into a pocket formed by the BH, funnel, and HB domains (Figure 3C; Figure S5). In the classic protein-centered view, interactions at the TL tip are primarily attributed to hydrophobic contacts, because the tip region is enriched in hydrophobic residues (Phe1084, Phe1086, Ala1087, Val1089, and Ala1090) (Figures 3B and 3C). In this view, only 2 direct hydrogen bonds are observed at this interface: one between HB residue Gln763 and TL residue His1085, and another between His1085 and the *β*-phosphate of the incoming ATP substrate (Figure 3B). However, our structure reveals an extensive water-mediated network bridging the TL tip with the funnel and HB domains. In total, 17 ordered water molecules are positioned at this interface, mediating interactions between the TL tip and 11 surrounding residues—2 from the HB domain and 9 from the funnel domain (Figures 3B and 3C). These interactions are predominantly formed through water-mediated hydrogen bonds involving the main chain of TL tip residues, effectively expanding the interaction network beyond the limited direct contacts previously observed (Figure 3C). In addition, 1 ordered water molecule (W19) coordinates 3 TL residues by interacting with the main-chain carbonyl groups of Phe1086 and Val1089, and the side chain of Thr1083, thereby stabilizing the fully closed conformation of the TL tip (Figure 3C). Together, these findings highlight the critical role of water molecules in stabilizing TL folding and mediating interactions between TL tip and other functional domains.

Category B waters (total 30 water molecules) mediate substrate recognition (see also above substrate recognition section) as well as communication with the anchor, cleft, and active-site domains, and the Rpb6 subunit (Figure 3B; Figures S5I–S5L); specifically, these water-mediated interactions establish extensive connections between TL and 9 cleft residues, 3 anchor residues, 9 active site residues, and one Rpb6 residue, respectively (Figure 3B; Figures S5I–S5L). These waters are important for mediating long-range communication between TL and other functional domains within RNA Pol II transcription machinery.

As for category C waters, we identified 7 additional water molecules (W7, W27, W29, W34, W36, W48, and W55) spanning the entire helix bundle interface between the TL (Val1066-Thr1083) and the BH (Gly823 to Ile848) (Figure 3D). These water-mediated interactions likely contribute to the stability and coupling of the TL/BH helices bundle.

### Extensive water-mediated interactions at proteinnucleic acid interfaces

As a key feature conserved in all multi-subunit RNAP ECs, tsDNA crosses over the BH domain and reaches the active site, where it forms a kinked conformation at the position between i + 1 and i + 2, with an angle of nearly 90°. The negatively charged phosphate groups get much closer in this kinked conformation. However, how this kinked conformation is maintained is not fully understood. Here, we reveal that the water molecules play an important role in maintaining the kinked conformation of tsDNA.

**Figure 4:**
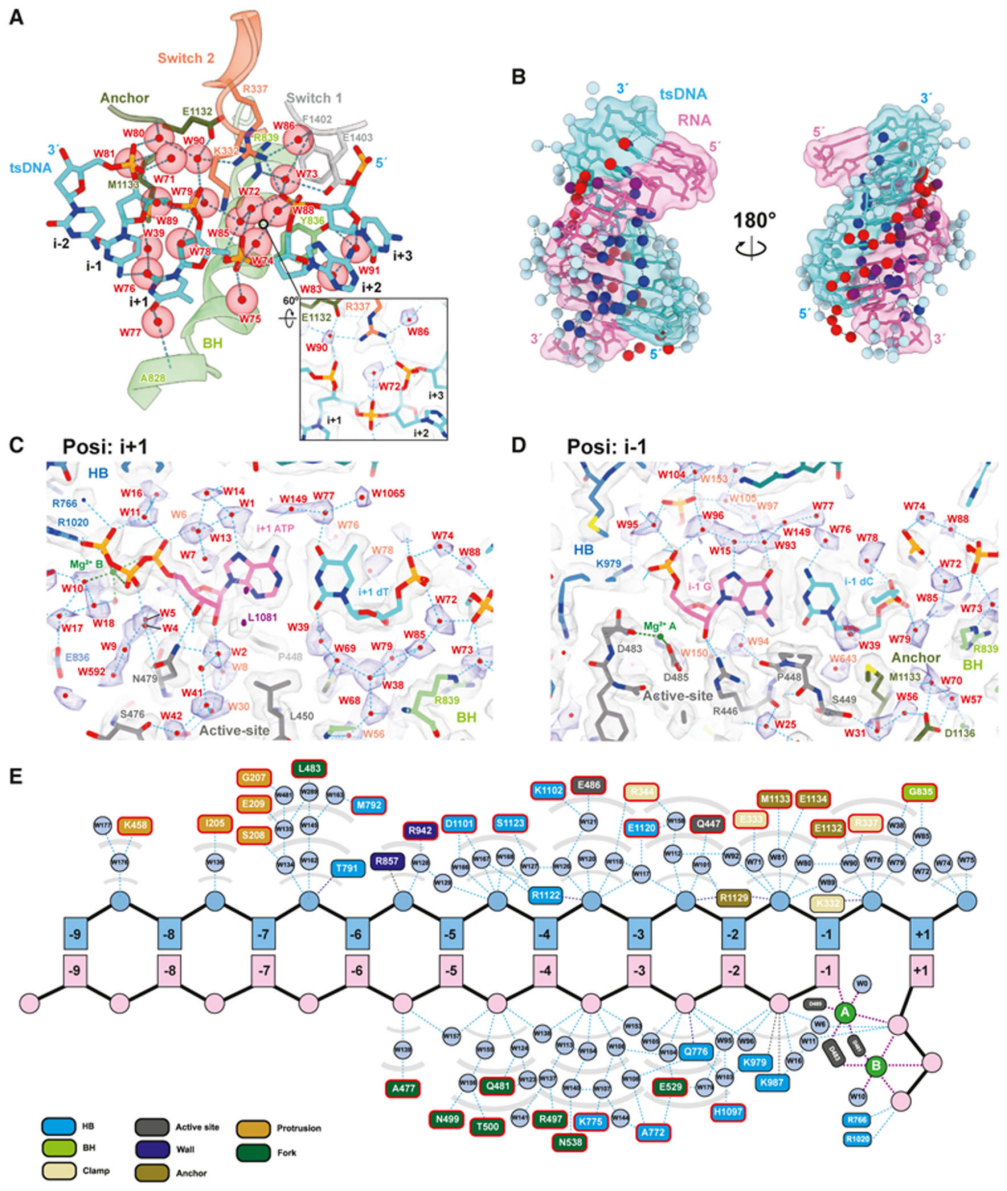
Water molecules stabilize kinked tsDNA, RNA-DNA hybrid, and protein-nucleic acids interfaces (A) Water-mediated interactions between the kinked tsDNA (cyan) and surrounding domains: BH (light green), switch loop 1 (silver), switch loop 2 (salmon), and anchor domain (olive). An inset shows the cryo-EM density of the kinked region, bent at ~90°, with the associated water molecule (density contoured at 5-σ). (B) Water molecules in the RNA-DNA hybrid region of RNA Pol II EC at the pre-catalysis state. Water molecules are grouped into 4 clusters according to their interaction partners within the hybrid region: interactions with the major groove (blue), minor groove (red), phosphodiester backbone (light blue), and 2′-OH of the RNA strand (purple). (C and D) Detailed views of water-mediated interactions at the i + 1 (C) and i – 1 positions (D). Cryo-EM densities are displayed as mesh contoured at 5-σ, and hydrogen bonds are indicated with blue dashed lines. (E) Cartoon representation of water-mediated interactions for the backbone of the RNA-DNA hybrid at the pre-catalysis state. Residues from different domains are color-coded as indicated. The residues forming water-mediated contacts with the RNA-DNA backbone are outlined with red borders (identified in this study). Hydrogen bonds and coordination bonds are depicted as blue and purple dashed lines, respectively.

We found extensive water-mediated interactions with the phosphodiester groups within the kinked region (i – 1, i + 1, and i + 2) (Figure 4A). A water molecule (W72) was found in the core of the kinked region, bridging the phosphate groups from i + 1 and i + 2 tsDNA and potentially stabilizing the kinked conformation (Figure 4A, close-up view). In addition, we found 19 waters (W39, W71–W81, W83, W85, W86, and W88–W91) that connect the kinked DNA with BH (Ala828, Tyr836, and Arg839), the switch loop 1 (Phe1402 and Glu1403), the switch loop 2 (Arg337 and Lys332), and the anchor domain (Glu1132 and Met1133) (Figure 4A).

In addition to the kinked tsDNA region, we also observed extensive water-mediated interactions in upstream RNA-DNA hybrid region (Figures 4B–4E; Figure S8). A hydration shell is identified to cover the RNA-DNA hybrid region (Figure 4B). Based on the locations and interactions, the water molecules can be categorized into 4 groups, including the backbone-interacting water molecules (63 waters, light blue sphere), major-groove-interacting water molecules (23 waters, blue sphere), minor-groove-interacting water molecules (24 waters, red sphere), and a group of water molecules involved in ribose discrimination (9 waters, purple sphere) (Figure 4B). Water molecules were found to form extensive hydrogen bonds with the polar groups of nucleobases on both RNA and tsDNA strands (Figures 4C and 4D). These waters form “water spines” that run along the minor and major grooves of the RNA-DNA hybrid, which are important for RNA-DNA hybrid stability^30,31^ (Figure 4B; Figure S8). The water spine observed within the RNA Pol II EC is similar to the previously reported crystal structure of a 9-mer RNA-DNA hybrid duplex (PDB: 421D).^30^ Notably, these ordered waters are mainly located between i+1 to i-6 base pairs, suggesting a potential role of these waters in stabilizing the RNA-DNA hybrid during transcription process (including initiation, elongation, and termination), in particular for short RNA-DNA hybrid during *de novo* transcription.

Intriguingly, in sharp comparison with the traditional view of RNA Pol II-nucleic acids interaction (mainly via direct interactions with positive residues), we now observed extensive water-mediated interactions at protein-nucleic acid interfaces involving a much broader range of RNA Pol II residues, which includes charged (both positive and negative), polar, and nonpolar residues (Figure 4E, residues with red rectangle outlines). We identified a total of 63 waters and 39 residues that are involved in protein-nucleic acids interactions (Figure 4E). Among them, only 8 residues are involved in direct interaction. These water molecules primarily mediate contacts between the phosphodiester backbones of RNA or DNA and RNA Pol II residues, forming hydration shells at the protein-nucleic acids interface in a sequence-independent manner (Figure 4E).

These water molecules at protein-nucleic acids interfaces likely act as molecular lubricants, facilitating the sliding of the nucleic acids scaffold along the binding interface and thereby promoting RNA Pol II translocation during transcription elongation. Two mechanisms may contribute to underlying processes causing this effect. First, displacing water molecules on the DNA surface requires less energy than breaking direct protein-DNA contacts, as shown by Overhauser Dynamic Nuclear Polarization (ODNP) studies.^32^ Second, water bridges enable a broader range of residues—including negatively charged, polar, and non-polar ones—to interact with nucleic acids beyond the typical positively charged Lys and Arg. Indeed, we identified 8 acidic residues that engage the RNA-DNA hybrid via water-mediated interactions, reducing the overall positive charges of nucleic acid binding surface and allowing smoother nucleic acid movement (Figure 4E).

### Water molecule dynamics during RNA Pol II catalysis

**Figure 5:**
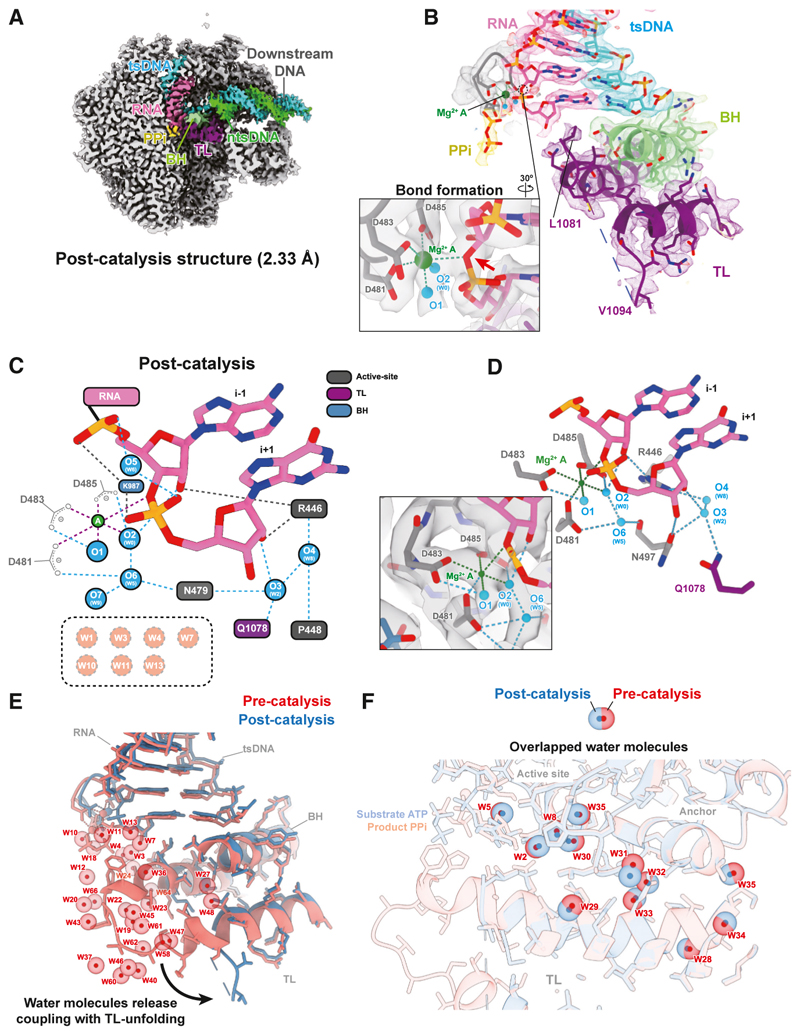
Cryo-EM structure of RNA Pol II EC captured at the post-catalysis state at 2.33 Å (A) Overview of the RNA Pol II EC in the post-catalysis state with a cross-sectional view. The product pyrophosphate (PPi) is shown in gold. Other elements are color-coded as indicated. (B) Cryo-EM density maps and corresponding atomic models of the active center in the post-catalysis structure. A close-up view highlights the newly formed phosphodiester bond, with the density map (contoured at 4-σ, contour level = 0.17). The red arrow marks the position of the new bond. Water molecules in the post-catalysis structure are shown in sky-blue sphere. (C) Interaction network of the newly incorporated nucleotide at the post-catalysis state. Water molecules identified in the post-catalysis structure are labeled O1–O7 (sky-blue sphere). Water molecules O2–O7 are conserved from the pre-catalysis state, with their corresponding identifiers (W0, W2, W5, W6, W8, and W9) indicated. A unique water molecule, O1, is observed exclusively in the post-catalysis state, where it coordinates with Mg^2+^ A. By contrast, water molecules W1, W3, W4, W7, W10, W11, and W13—present in the pre-catalysis state—are absent post-catalysis and shown as transparent red spheres. Residues from different domains are color-coded as indicated. The representations of hydrogen bonds and coordination bonds are consistent with those in Figure 2A. (D) Detailed structural view of the interactions involving the newly incorporated nucleotide. A close-up view shows the density maps of Mg^2+^ A coordination, contoured at 4-σ. (E) Structural comparison of water molecules associated with TL folding and unfolding between the pre-catalysis (salmon) and post-catalysis (steel blue) states. Water molecules that interact with the folded TL tip in the pre-catalysis state are absent in the post-catalysis state due to TL un-folding. (F) Superposition of pre-catalysis (salmon) and post-catalysis (steel blue) structures highlighting the overlapping water molecules positioned at the interface between the TL, active site domain, and anchor domain.

To gain insight into the roles of waters during RNA Pol II NTP addition reaction, we determined a cryo-EM structure of the RNA Pol II EC captured at a transient post-catalysis state at 2.33 Å resolution, by allowing NTP addition on cryo-EM grid (Figures 5A and 5B; Figure S9). Examination of the active center in this structure revealed well-defined densities corresponding to the newly formed phosphodiester bond between the RNA primer and the incoming substrate (Figure 5B inset, red arrow), as well as the PPi product (Figure 5B). Interestingly, the PPi migrates away from its original position shared by metal B and *β*- and *γ*-phosphate of the NTP in pre-catalysis state, suggesting a transition pathway toward release via the secondary channel. In contrast to the well-defined density of metal B in pre-catalysis state, the density of metal B in post-catalysis state is less defined, reflecting its dynamic nature. TL adopts an unfolded conformation in the post-catalysis state (Figure 5B). Notably, Leu1081, a key residue involved in stabilizing the incoming sub-strate NTP in the pre-catalysis state, remains detectable in the post-catalysis structure (Figure 5B). The nucleic acid scaffold remains the same as in the pre-translocation configuration (Figure 5B).

In the post-catalysis state, we identified over 712 ordered water molecules. To further investigate the functional and structural roles of water molecules within the RNA Pol II EC during catalytic reaction, we compared water distribution patterns between pre- and post-catalysis structures. While most water molecules (>92%, 660 overlapped water out of 712 total water in post-catalysis structure) occupy similar positions in both states, those located at the active site show markedly distinct distributions (Figures 5C and 5D; Data S3). Based on these differences, we classified the waters into 2 major categories: catalytic-state-specific waters (class 1) and structural waters (class 2).

Class 1 waters are specific to a particular catalytic state and are primarily localized near the active site. Accompanying nucleotide addition and TL unfolding, a total of 28 water molecules (W3, W4, W7, W10–W13, W18–W20, W22–W24, W27, W36, W37, W40, W43, W45–W48, W58, W60–W62, W64, and W66) are absent in the post-catalysis structure, underscoring their functional importance in stabilizing the folded TL and its interaction with other active center motifs in the pre-catalysis state (Figures 5C–5E; Video S2). Following catalysis, we observed the appearance of a unique Mg^2+^-A-coordinated water molecule (O1) exclusively present in the post-catalysis structure. This water replaces the coordination that is previously provided by the *α*-phosphate oxygen of the NTP in the pre-catalysis state. As a result, 2 waters (O1 and W0) coordinate with Mg^2+^ A in the post-catalysis state (Figures 5B–5D).

Class 2 waters are present in both pre- and post-catalysis structures (Figure 5F; Data S3). Notably, 660 of 712 identified water positions in the post-catalysis structure (over 92%) over-lap with corresponding water positions in the pre-catalysis structure within a 1.5 Å threshold, highlighting their remarkable positional consistency. This strong conservation underscores the role of structural water molecules as integral components of RNA Pol II EC architecture. These waters contribute to both subunit-subunit interactions and protein-nucleic acid interfaces.

Remarkably, despite the nucleotide sequence variation between the 2 states, a persistent hydration shell—particularly clusters of ordered water molecules—is observed along the i + 1 to i – 6 base pairs in both structures (Figures S8I and S8J). While individual water molecules interacting with major-groove and minor-groove edges may shift slightly to accommodate different base-pair functional groups, the overall hydration pattern remains conserved (Figures S8I and S8J). This sequence-independent clustering suggests that water-mediated interactions in the i + 1 to i – 6 region are an intrinsic structural feature of RNA Pol II’s engagement with the RNA-DNA hybrid. These interactions likely play essential roles in precise positioning of the hybrid and substrate during catalysis and may also help stabilize short RNA-DNA hybrid during *de novo* transcription, thereby reducing abortive transcription. Similarly, the pattern of water-mediated interactions within the kinked tsDNA region is highly conserved across both states.

## Discussion

### Water molecules are essential for proton transfer during RNA Pol II catalysis

High-resolution structures with water molecules also offer mechanistic insights into proton transfer during RNA Pol II catalysis. The absolutely conserved TL residue His1085 was hypothesized to function as a proton donor, as it is the only RNA Pol II residue that directly contacts the *β*-phosphate of ATP.^5,8,33^ However, genetic and biochemical results of this conserved residue are perplexing: Several substitutions at this position (e.g., H1085A, H1085D, H1085N, or H1085F) are lethal in yeast, whereas certain substitutions (e.g., H1085Q or H1085L) remain viable, albeit with a 2- to 10-fold reduction in transcription elongation rate in these mutants.^34,35^ These results suggest that the proton-donating capacity of His1085 is not strictly essential. Importantly, the viability of H1085Q and H1085L mutants suggests the presence of other proton donors for RNA Pol II catalysis in these H1085 mutants.

**Figure 6:**
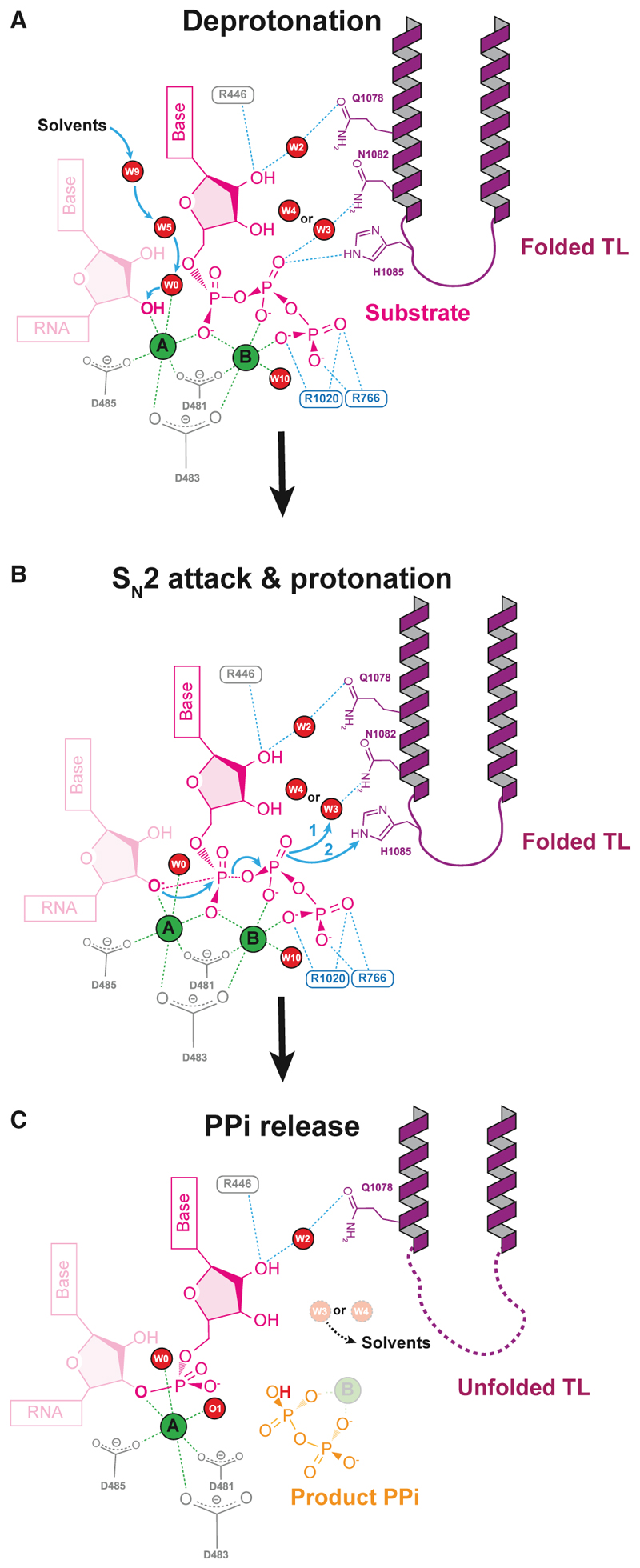
Substrate recognition and catalytic mechanism of RNA Pol II EC in water-integrated view Proposed RNA Pol II catalytic mechanism, including deprotonation of the RNA primer 3′-OH (A), S_N_2 attack and protonation of PPi (B), and PPi release (C). During the deprotonation step (A), the proton of 3′-OH of the RNA primer is transferred through a water chain (W0-W5-W9) that connects to the bulk solvent. In S_N_2 attack step (B), Water molecules W3 and W4 are positioned to function as proton donors. Pathway “1” represents the water-mediated protonation route involving W3/W4, whereas pathway “2” denotes the alternative His1085-mediated route. In the post-catalysis state (C), formation of the PPi (orange) product is coupled to TL unfolding (depicted by purple dashed lines). Electron transfer pathways are illustrated by blue arrows.

Our high-resolution structures now provide a clear structural explanation for this observation. We identify 2 ordered water molecules (W3 and W4) that directly engage the *β*-phosphate of the incoming NTP and are geometrically positioned to participate in proton transfer. These observations highlight the functional importance of these waters as proton donors in wild type (WT) as well as H1085L and H1085Q mutants. In addition to these water-mediated interactions, we also unambiguously confirmed the direct interaction between His1085 and *β*-phosphate (Figure 6; see below).

Leveraging our high-resolution cryo-EM structures and previous studies,^5,12,23,36,37^ we now propose a water-mediated S_N_2 catalytic model for the RNA Pol II EC that explicitly demonstrates the critical roles of water molecules (Figure 6; Video S2). Prior to catalysis, water molecules mediate key interactions that support TL folding and substrate recognition (Figures 2A, 3B, and 6A). The base, sugar and phosphate moieties of the incoming NTP are recognized via both direct and water-mediated interactions (Figures 2A and 6A). During catalysis, a chain of water molecules plays critical roles in deprotonating 3′-OH. We found that water molecule W0 coordinates with Mg^2+^ A and acts as a proton acceptor, abstracting a proton from the 3′-OH of RNA primer. This deprotonation step reduces the active energy barrier for S_N_2 nucleophilic attack on the *α*-phosphate of the NTP. W0 transfers the proton to the solvent via a water chain relay involving W5 and W9, which is consistent with prior computational studies showing that this pathway has the lowest energy barrier (Figure 6A).^33^

As the reaction proceeds, a pentavalent phosphate intermediate is formed (Figure 6B). The electron transfer pathway is illustrated by blue arrows (Figure 6B). Consequently, a new phosphodiester bond is formed between the 3′-RNA primer and the α-phosphate of the substrate NTP, breaking the bond between the α- and β-phosphates, extending the RNA primer by one nucleotide, and releasing PPi (Figure 6C). Our structures also clarify the long-standing question of how PPi is protonated. We reveal that water molecules (W3 and/or W4) and/or His1085 provide redundant protonation pathways, ensuring catalytic robustness These water molecules serve as proton donors to ensure that catalysis proceeds, even in the absence of His1085 (when His1085 is mutated).

### Water molecules are evolutionarily conserved among prokaryotic and eukaryotic transcription machineries

Our work revealed the essential roles of waters in RNA Pol II transcription. This also raises an intriguing question: whether these functionally important waters are evolutionally conserved across different RNA polymerases among prokaryotic and eukaryotic transcription machineries. Intriguingly, we compare water peaks identified from RNA Pol II EC and *E. coli RNAP* complex (please see Mueller and Darst^22^). Strikingly, as shown in Figure S10 and Data S3, we are able to identify over 230 common waters that are shared among different structures within the cutoff 1.5 Å. Key water molecules that are involved in deprotonation and protonation steps during catalytic reaction are strictly conserved between *E. coli* RNAP and *S. cerevisiae* RNA Pol II (Figure S10). These waters include W0/W5/W9 water chains, proposed to deprotonate 3′-OH, and W3,—proposed to act as a proton donor for PPi protonation during the nucleotide incorporation reaction (Figure S10). Another potential proton donor, water molecule W4, is absent in *E. coli*, which may be attributed to the substitution of the TL residue Asn1082 in *S. cerevisiae* RNA Pol II with Arg933 in *E. coli* RNAP (Figure S10). The majority of water molecules for substrate recognition are also highly conserved (11 out of 13 common waters, Figure S10). These striking findings further highlight that these functional water molecules are evolutionarily conserved integral components of transcription machineries and play critical roles in the structure and function of transcription.

### Conclusions

Our high-resolution RNA Pol II EC structures at distinct stages of nucleotide addition resolve long-standing ambiguities in active-site architecture, such as precise substrate and metal ion coordination within a fully assembled catalytic center. More importantly, our study reveals important roles of water molecules in RNA Pol II catalysis during nucleotide addition, substrate recognition, and TL folding. Unlike a canonical protein centered view, we now uncover extensive and previously underappreciated water-mediated interaction networks across the transcription machinery. They also serve as integral structural elements, stabilizing kinked DNA, mediating subunit interfaces, and facilitating protein-nucleic acids interactions. Critically, our findings redefine the nature of RNA Pol II-nucleic acid contacts, revealing that water bridges enable interactions involving a broad spectrum of residue types—including negatively charged and nonpolar residues—beyond the canonical positively charged ones. Such knowledge is highly relevant to many other nucleic acid enzymes or nucleic acid binding proteins. The concept of water as a molecular lubricant may represent a general mechanism for facilitating nucleic acid translocation across diverse nucleic acid processing enzymes and motor proteins.

Together with the study of *E. coli RNAP*, we revealed that these functionally important waters in RNAP catalysis are evolutionarily conserved between prokaryotic and eukaryotic multi-subunit RNA polymerases. These observations highlight the fundamental importance of waters in transcription. Our work provides a foundational framework for future MD simulation and time-resolved structural analyses aimed at probing the dynamic roles of ordered and disordered water and ions during transcription.

### Limitations of the study

Our sub-2 Å cryo-EM structures identify 2 ordered water molecules positioned toward the *β*-phosphate of the incoming NTP. Based on their geometry and proximity, these waters provide a structurally plausible proton donor pathway operating in parallel with His1085. It is worthy to note that previous mutation and biochemical studies as well as current structural studies cannot completely exclude a potential contribution from His1085 as a proton donor, in addition to these waters in WT RNA Pol II. At the physiological pH, His1085 can exist in both protonated (charged) and deprotonated (neutral) states, both of which can form hydrogen bonds with the phosphate of substrate. The protonated state of His1085 would be readily to serve as a proton donor during S_N_2 reaction. Accordingly, we propose a parallel model for protonation transfer in which water-mediated and His1085-mediated pathways may coexist. However, this study cannot quantify the relative contributions of water-mediated and His1085-mediated proton transfer in WT RNA Pol II. Thus, the precise role and relative contribution of His1085 in the proton transfer step remain an open question that will require future biochemical and computational investigations to resolve.

## Resource Availability

### Lead contact

Correspondence and requests for materials should be addressed to Dong Wang (dongwang@ucsd.edu).

### Materials availability

Materials are available from Dong Wang upon request under a material transfer agreement.

## Star★Methods

### Key Resources Table

**Table:** 

REAGENT or RESOURCE	SOURCE	IDENTIFIER
Bacterial and virus strains		
BL21 (DE3) *E. coli*	NEB	Cat#C2527
Chemicals, peptides, and recombinant proteins		
ATP Solution (100 mM)	Thermo Fisher Scientific	Cat#R0441
CTP Solution (100 mM)	Thermo Fisher Scientific	Cat#R0451
UTP Solution (100 mM)	Thermo Fisher Scientific	Cat#R0471
GTP Solution (100 mM)	Thermo Fisher Scientific	Cat#R0461
Biotin-labeled lipid (16:0 Biotinyl Cap PE)	Avanti	Cat#870277
DOPC (18:1 (Δ9-Cis) PC)	Avanti	Cat#850375
Streptavidin from *Streptomyces avidinii*	Sigma	Cat#S0677
Holy carbon grids (Quantifoil copper 2/1, 300 mesh)	Quantifoil	Cat#Q350CR1-2nm
Teflon well	Ma et al.^21^	N/A
Deposited data		
1.96 Å high-resolution map of the Pol II EC at pre-catalysis state (with Elf1)	This study	EMDB: EMD-55239
Local refinement map of the nucleic acid scaffold at pre-catalysis state (with Elf1)	This study	EMDB: EMD-55240
2.26 Å high-resolution map of the Pol II EC at pre-catalysis state (Elf1-free)	This study	EMDB: EMD-53053
A composite map of the global Pol II EC at pre-catalysis state (Elf1-free)	This study	EMDB: EMD-53057
Local refinement map of nucleic acid scaffold at pre-catalysis state (Elf1-free)	This study	EMDB: EMD-53064
Local refinement map of Rpb4/7 at pre-catalysis state (Elf1-free)	This study	EMDB: EMD-53056
Local refinement map of Rpb9 at pre-catalysis state (Elf1-free)	This study	EMDB: EMD-53060
Local refinement map of Rpb12/Wall at pre-catalysis state (Elf1-free)	This study	EMDB: EMD-53063
Local refinement map of Jaw/Rpb9 at pre-catalysis state (Elf1-free)	This study	EMDB: EMD-53062
2.33 Å* *high-resolution map of the Pol II EC at post-catalysis state (Elf1-free)	This study	EMDB: EMD-54374
1.96 Å* *high-resolution model of the Pol II EC at pre-catalysis state (with Elf1)	This study	PDB: 9SV6
2.26 Å* *high-resolution model of the Pol II EC at pre-catalysis state (Elf1-free)	This study	PDB: 9QEB
2.33 Å* *high-resolution model of the Pol II EC at post-catalysis state (Elf1-free)	This study	PDB: 9RYB
Experimental models: Organisms/strains		
*S. cerevisiae:* Strain background: BJ926 (TAP-tag on Rpb3)	Wang et al.^5^	N/A
Oligonucleotides		
Biotinylated template strand DNA (tsDNA): 5’-/BiotinTEG/ TTT TTT GAT ATT TTT GGA TCC CGC TCT GCT CCT TCT CCC ATC CTC TCG ATG GCT ATG AGA TCA ACT AGG AAT TC-3’	IDI	N/A
Biotinylated non-template strand DNA (ntsDNA): 5’-/BiotinIEG/ TTT TTA TGT ATT AAT GAA TTC CTA GTT GAT CTC ATA GCC CAT TCC TAC TTG GGA GAA GGA GCA GAG CGG GAT CC-3’	IDI	N/A
3’-deoxy RNA (for pre-catalysis): 5’-AUC GAG AG/3’dG	IDI	N/A
regular RNA (for post-catalysis): 5’-AUC GAG AGG	IDI	N/A
Recombinant DNA		
Plasmid: pGEX-6P-1_Elf1	Sarsam et al.^38^	N/A
Plasmid: Rpb4/7	Wang et al.^5^	N/A
Software and algorithms		
EPU	Ihermo Fisher Scientific	https://www.thermofisher.com/us/en/home/electron-microscopy/products/software-em-3d-vis/epu-software.html
cryoSPARC (v4.5.3)	Punjani et al.^39^	https://cryosparc.com/
UCSF ChimeraX (v1.8)	Goddard et al.^40^	https://www.rbvi.ucsf.edu/chimerax/
UCSF Chimera (v1.17)	Pettersen et al.^41^	https://www.cgl.ucsf.edu/chimera/
PHENIX (v1.21)	Adams et al.^42^	https://phenix-online.org/
COOT (v0.9.8)	Emsley et al.^43^	https://www2.mrc-lmb.cam.ac.uk/personal/pemsley/coot/
Segger(v2.9.1)/SWIM	Zhang et al.^44^	https://github.com/gregdp/segger/tree/master
GROMACS (v2022.5)	Abraham et al.^45^	https://www.gromacs.org/

### Experimental Model and Study Participant Details

#### Plasmids and strains

*Saccharomyces cerevisiae* strains expressing endogenously TAP-tagged RNA polymerase II were cultured in 2×YPD medium at 30°C.

Plasmids encoding Rpb4/7 and Elf1 were transformed into *E. coli* BL21 (DE3) cells and expressed recombinantly in standard LB medium.

### Method Details

#### Protein purification and complex assembly

*Saccharomyces cerevisiae* 12-subunit RNA polymerase II (Pol II) was purified following procedures described previously.^5,46^ To assemble the complete 12-subunit Pol II, the purified 10-subunit Pol II was mixed with the recombinant Rpb4/7 subcomplex *in vitro* and subjected to gel filtration chromatography purification. Fractions containing the fully assembled 12-subunit Pol II were collected for further structural analysis.

The biotinylated Pol II elongation complex was assembled *in vitro* as previously reported.^21^ Briefly, all RNA and DNA oligonucleotides used in this study were PAGE-purified and purchased from Integrated DNA Technologies (IDT). The sequences used to form the elongation complex were: Biotinylated template strand DNA (tsDNA): 5′-/BiotinTEG/ TTT TTT GAT ATT TTT GGA TCC CGC TCT GCT CCT TCT CCC ATC CTC TCG ATG GCT ATG AGA TCA ACT AGG AAT TC-3′; Biotinylated non-template strand DNA (ntsDNA): 5′-/BiotinTEG/ TTT TTA TGT ATT AAT GAA TTC CTA GTT GAT CTC ATA GCC CAT TCC TAC TTG GGA GAA GGA GCA GAG CGG GAT CC-3′; 3′-deoxy RNA (for pre-catalysis): 5′-AUC GAG AG/3′dG; regular RNA (for post-catalysis): 5′-AUC GAG AGG.

To assemble the nucleic acid scaffold, the RNA and template-strand DNA (tsDNA) were first mixed and annealed. For pre-catalysis samples, a 3′-deoxy RNA was used to prevent substrate incorporation, whereas for post-catalysis samples, a regular RNA was used to permit catalysis. Pol II was then added to assemble the elongation complex (EC), followed by addition of the non-template DNA (ntsDNA) strand to yield the fully assembled EC. Complex formation was carried out in elongation buffer [20 mM Tris-HCl (pH 7.5), 40 mM KCl, 10 mM MgCl_2_, and 5 mM DTT]. The final concentrations of components in the assembled EC were 1 μM Pol II, 1.2 μM tsDNA, 1.5 μM ntsDNA, and 1.2 μM RNA. To improve resolution of the pre-catalysis state, 5 μM Elf1 was added to one pre-catalysis sample, while a parallel sample assembled with Pol II alone (without Elf1) was prepared for comparison.

Reactions were initiated by substrate addition. For pre-catalysis samples, 5 mM ATP was added to the fully assembled EC, followed by incubation at room temperature for 10 min prior to cryo-EM grid preparation. For post-catalysis samples, the “substrate gradient grid” method was applied by adding 10 mM NTP to initiate the reaction. Detailed procedures for this method are described in the cryo-EM sample preparation section.

#### Cryo-EM sample preparation

The optimized protocol for preparing mspSA affinity-enriched grids is outlined as follows^21^: DOPC (18:1 (Δ9-Cis) PC, Avanti) and biotin-labeled lipid (16:0 Biotinyl Cap PE, Avanti) are mixed in a 9:1 ratio to reach a final concentration of 1 mg/mL. Prepare a streptavidin solution (streptavidin from *Streptomyces avidinii*, Sigma) at a concentration of 0.01 mg/mL in a buffer consisting of 20 mM HEPES (pH 7.5) and 150 mM NaCl.^47^ Add 30 μL of the streptavidin solution to a Teflon well (on ice), and then carefully lay 0.7 μL of the lipid mixture over the surface of the streptavidin solution. Enclose the Teflon block in a humidity chamber containing a small amount of Milli-Q water at the bottom of the chamber. Seal the lid of the chamber with Vaseline and incubate overnight at room temperature. After 6 hours, carefully place the carbon side of holy carbon grids (Quantifoil copper 2/1, 300 mesh) onto the surface of the Teflon well (without prior glow discharge) for 2 minutes to allow for lipid monolayer transfer. Wash the grid with three drops (120 μL) of sample buffer to remove unbound streptavidin. Add 4 μL of the Pol II EC sample to affinity grids and incubate for approximately 5 minutes. After incubation, remove the excess sample solution using filter paper. Subsequently, plunge-freeze the grids using the Thermo Fisher Vitrobot IV, applying the following parameters: 3.5 seconds blotting time, -15 blotting force, at 4°C and 100% humidity with adding 3.5 μL of sample buffer. The blotting force should be optimized to achieve optimal vitrification results.

To capture the post-catalysis structure of the Pol II EC, the PoI II EC was first applied to the affinity grids and incubated for 10 minutes. Grids were washed with 50 μL fresh buffer, and excess solution was removed using filter paper. The grids were then transferred to Thermo Fisher Vitrobot IV. 3 μL fresh buffer was added to each grid and subsequently 0.2 μL of 10 mM NTP substrate was introduced onto the grid at the top side immediately before blotting, generating a “Substrate Gradient Grid (SGG)”. The regions selected for data collection were marked on the grid (Figure S9).

#### Data collection and processing

Initial screening to obtain integral grids with well-formed monolayers and particles was conducted using a 200 kV Thermo Scientific™ Glacios™ transmission electron microscope. Cryo-EM data were collected using a FEI Titan Krios operating at 300 kV, equipped with a Gatan K3 with GIF Quantum camera (at eBIC, Diamond) or Falcon 4 with GIF Quantum camera (OPIC, STRUBI, University of Oxford). Data acquisition was performed automatically using EPU software, with defocus values ranging from -0.5 to -2.5 μm. The imaging pixel size for data collection was 0.829 Å for pre-catalysis Pol II EC (Bio-Quantum K3), 0.825 Å for post-catalysis Pol II EC (Bio-Quantum K3) and 0.932 Å for pre-catalysis Pol II EC associated with Elf1 (Falcon 4 with SelectrisX), and the total dose applied was 50 electrons per Å^2^, distributed across 50 frames. A total of 10,599 images were collected for pre-catalysis Pol II EC, 18,913 images for post-catalysis Pol II EC and 16,482 images for pre-catalysis Pol II EC associated with Elf1.

All datasets were processed via CryoSPARC (v4.5.3).^39^ For pre-catalysis Pol II EC without Elf1, raw micrographs were imported, followed by motion correction and calculation of the contrast transfer function (CTF). The resulting micrographs were manually curated to exclude images with poor quality, (such as CTF fit resolution: <4Å; Relative ice thickness: 1< values < 1.1; Total full-frame motion distance (pixels): < 60 and astigmatism: < 5000). All micrographs were then subjected to automatic particle picking (Blob picking), with particle diameters ranging from 130 Å to 170 Å, to generate initial 2D templates. Following several rounds of 2D classification, particles with good 2D class averages were selected for Topaz training^48,49^ using the complete dataset. After further 2D classification, high-quality particles with distinct 2D class averages were merged, and duplicate particles were excluded. The output particles were used for ab-initio reconstruction, followed by hetero-refinement. Particles selected from good 3D classes after hetero-refinement were used for Topaz training. After multiple rounds of Topaz training on different particle sets, high-quality particles obtained from both Blob picking and Topaz training were merged to eliminate duplicates. These refined particle sets were then used for ab-initio reconstruction, followed by hetero-refinement. The best 3D class was selected for defocus refinement, global CTF refinement, and non-uniform refinement. To minimize the influence of the dynamic region of the Rbp4/7 stalk and enhance the resolution, a truncated mask without Rbp4/7 was applied for local refinement, resulting in a 2.26Å core structure of the EC complex. To obtain a complete 12-subunit Pol II EC structure, the five flexible regions were subjected to further refinement. The five flexible regions include the nucleic acid scaffold, Rpb4/7, Rpb9, Rpb12/wall, and Jaw/Rpb9. Focused masks were employed for 3D classification processing on the flexible regions, then local or homogeneous refinement jobs were applied to refine each local region. The final complete map was generated by compositing maps of core Pol II and the flexible regions. For the post-catalysis Pol II EC, a processing strategy similar to that used for the pre-catalysis complex was applied. Following motion correction, contrast transfer function (CTF) calculation, particle picking, 2D classification, ab-initio reconstruction, hetero-refinement, and homo-refinement, 3D classification with a solvent mask identified 6 out of 15 classes exhibiting TL unfolding and bond formation. Particles from these classes were selected for further non-uniform refinement, resulting in a final 2.33 Ådensity map. For pre-catalysis Pol II EC associated with Elf1, the same processing strategy as post-catalysis Pol II EC was used, except that 3D classification with a solvent mask identified 2 out of 12 classes exhibiting TL folding. Particles from these classes were selected for further non-uniform refinement, followed by reference-based motion correction and an additional round of non-uniform refinement, resulting in a 1.96 Å density map.

In all cases, the resolution was determined by gold-standard Fourier shell correlation (FSC). The local resolution estimation was calculated in Chimera^41^ based on the output maps from CryoSPARC.

#### Atomic model building, refinement, and validation

The initial models for the 12-subunit *S. cerevisiae* RNA Pol II were derived from previously published crystal structures (PDB: 2E2H^5^ and 8U9R^12^). For Elf1 in the pre-catalysis structure, the initial model was obtained from the published cryo-EM structure (PDB: 8TVY^38^). The resulting model was rigid-body fitted into the cryo-EM density map using ChimeraX UCSF (v1.8).^40^ Refinement of the fitted model was conducted in PHENIX (v1.21)^42^ using the phenix.real_space_refine program. The refined model was subsequently manually adjusted in COOT (v0.9.8).^43^ Model validation was carried out with MolProbity.^50^ All structural figures were rendered using ChimeraX UCSF (v1.8).^40^

#### Identification of water and ion molecules

Based on previously established methodologies, we developed a systematic workflow to accurately identify and model water and ion molecules in our cryo-EM structures. This workflow comprises three primary steps: candidate peak identification, peak validation, and molecular assignment of water and ions (Figure S6A). Initially, we segmented the sharpened cryo-EM density maps using the Segger plugin in UCSF Chimera. Potential peaks corresponding to water or ion molecules were identified by the automated peak identification program SWIM^44^ and additional manual peak selection.

To ensure accuracy and remove spurious noise peaks, we applied two stringent validation thresholds: a minimum density threshold of 4-σ, and a Q-score cutoff of 0.7. The Q-score, a quantitative metric designed to evaluate the local fit of atomic models within cryo-EM density maps, has been previously established and widely utilized in recent high-resolution cryo-EM studies to validate the accurate placement of water and ion molecules.^15,51,52^ To further enhance the robustness of our validation, we employed cross-validation using two independent sharpened half-maps (half-A and half-B) in conjunction with the full map. Each peak was rigorously cross-checked to ensure that its Q-score exceeded 0.7 consistently across all three maps. This cross-validation strategy is conceptually analogous to anomalous signal validation in X-ray crystallography and has recently been successfully applied in cryo-EM studies to robustly confirm water and ion placements.^15,44^ For detailed Q-score value of individual water and ion molecules, please refer to Data S1.

Validated peaks were subsequently evaluated using our molecular assignment scoring system to differentiate between water molecules and specific ion types. This assignment scoring system calculates the probability that each peak corresponds to one of four possible molecular species *S* ∈{H_2_O, Na^+^, K^+^, Mg^2+^} (Figure S6A). Each candidate peak was ultimately assigned the molecular identity corresponding to the highest calculated score. The overall score for each molecular identity was derived from the product of four distinct scoring functions: distance-scoring function $\left(f_{distance}^{S}\right)$, the coordination-number scoring function $\left(f_{counts}^{S}\right)$, the charge-scoring function $(f_{charge}^{\text{S}})$, and the clash function $(f_{clash}^{\text{S}})$.

(1) Distance-score $f_{distance}^{S}$

For each candidate site we measure all of its *n* polar-contact distances (*d*_*i*_) to surrounding polar atoms. We then score each distance with a flat-top Gaussian kernel, which gives perfect score inside an “ideal” coordination distance range (*μ*_*S*_ ± *h*_*S*_) and smoothly decays to zero over a tail of length */* (default: 0.2 Å). The *μ*_*S*_ and *h*_*S*_ respectively represent the mean distance and half width of coordination distance range for each molecule species (*S*). The theoretical distance ranges for the distinct molecule coordination were input as: 2.9 ± 0.5 Å for water molecules, 2.0 ± 0.2 Å for Mg^2+^, 2.5 ± 0.1 Å for Na^+^, and 2.9 ± 0.3 Å for K^+^. The scoring function for each distance is defined as:

$$k_{i}^{S}\left(d_{i}\right)=\{\begin{array}{cc} 1, & \left|d_{i}-\mu_{S}\right|\leq h_{S}\\ \exp\left[-\frac{1}{2}{\left(\frac{\left|d_{i}-\mu_{S}\right|-h_{S}}{\sigma }\right)}^{2}\right], & h_{S}<\left|d_{i}-\mu_{S}\right|<h_{S}+I\\ 0, & \left|d_{i}-\mu_{S}\right|\geq h_{S}+I \end{array}$$

The Gaussian standard deviation (*σ*) is chosen so that the kernel falls to 0.5 at the midpoint of the tail:

$$\sigma =\frac{1/2}{\sqrt{2ln2}}$$

The average distance-score is:

$$f_{distance}^{S}=\frac{1}{N}\sum_{i=1}^{N}k_{i}^{S}\left(d_{i}\right)$$

(2) Contact-counts score $f_{counts}^{S}$

We utilized a linear contact-count score to quantify how the total number of polar contacts “*n*^*S*^” at a candidate site agrees with the expected coordination number of each species. For water molecules, the ideal maximum number of polar contacts is four. Accordingly, the scoring function for water assigns a maximum score of 1 when $n_{P}^{water}$ is less than or equal to 4. If the number of detected polar contacts exceeds four, a penalty is applied to reflect the reduced likelihood of accurate water assignment. The Δ is a soft-cutoff width that governs the rate at which the score decays (default Δ= 8). The complete coordination-scoring function for the water molecule is defined as:

$$f_{counts}^{water}(n)=\left\{\begin{array}{cc} 1, & 0<n_{P}^{water}\leq c_{max}^{water}\\ 1-\frac{n_{P}^{water}-c_{max}^{water}}{\text{Δ}}, & c_{max}^{water}<n_{P}^{water}\leq 6\\ 0, & n_{P}^{water}>6 \end{array}\right\}$$

In contrast to water, ion placement requires a minimum amount of coordinating oxygen to be chemically plausible. Therefore, if the coordination number of oxygen $\left(n_{O}^{ion}\right)$ falls below a defined minimum threshold $\left(c_{min}^{ion}\right)$, the candidate site is penalized accordingly. To model this behavior, we applied a scoring function that assigns low values for insufficient coordination and gradually increases as the number of contacts approaches and exceeds $c_{min}^{ion}$. The minimum coordination number $c_{min}^{ion}$ was set to four for all tested ion species. The maximum expected coordination number $c_{min}^{ion}$ was set to six for Na^+^ and Mg^2+^, and eight for K^+^. The full formulation of the coordination-number scoring function for ion candidates is as follows:

$$f_{counts}^{ion}(n)=\left\{\begin{array}{cc} \frac{n_{O}^{ion}}{c_{min}^{ion}}, & 0<n_{O}^{ion}\leq c_{min}^{ion}\\ 1, & c_{min}^{ion}<n_{O}^{ion}\leq c_{max}^{ion}\\ 0, & n_{O}^{ion}>c_{max}^{ion} \end{array}\right\}$$

(3) Electrostatic-Charge Term $f_{counts}^{S}$

The local electrostatic environment is another critical consideration in molecule assignment. We defined the nitrogen from the side chain of residues Arg and Lys as the basic atoms and oxygen from the side chain of residues Asp and Glu, and phosphate groups on nucleic acid backbone as the acidic atoms. The set of contact for charged atoms was denoted as *C*_*positive*_ and *C*_*negative*_. For water molecules, given their ability to form polar interactions with both positively and negatively charged groups, the charge-scoring function always returns a score of “1”. In contrast, metal ions cannot form stable coordination bonds with positively charged groups. Therefore, if a positively charged group is detected within the given distance value of 4 Å, the charge-scoring function scores “0”. In addition, the coordination of Mg^2+^ ion requires more than one negative charge group. Taken together, the complete charge-scoring function is defined as:

$$f_{charge}^{S}=\{\begin{array}{cc} 1, & S={\text{H}}_{2}\text{O},\\ \{\begin{array}{c} 1,\\ 0, \end{array} & \begin{array}{l} S\in \left\{Na^{+},K^{+}\right\},C_{positive}=0\\ S\in \left\{Na^{+},K^{+}\right\},C_{positive}>0 \end{array}\\ \{\begin{array}{c} 1,\\ 0, \end{array} & \begin{array}{l} S\in \left\{Mg^{2+}\right\},C_{positive}=0\wedge C_{negative}>0\\ S\in \left\{Mg^{2+}\right\},C_{posilive}>0\vee C_{negative}=0 \end{array} \end{array}$$

(4) Clash Term $f_{clash}^{S}$

To avoid potential clash, we used a penalty scoring function for multiple “too-short” contacts. The polar contact within distance of (*μ*_*S*_ *– h*_*S*_ *– l*) was defined as a “too-short” contact.

$$\begin{array}{c} n_{short}^{S}=\text{\#}\left\{i:d_{i}<\mu_{S}-h_{S}-l\right\}\\ f_{short}^{S}=\cos\left(min\left(\frac{n_{short}^{S}}{\alpha },\frac{\pi }{2}\right)\right) \end{array}$$

Regarding the ion assignment, main-chain nitrogen contacts (atom name “N”) within the filter window are rarely compatible with metal coordination. We count “$n_{N}^{ion}$ of these and apply the same half-cosine form:

$$f_{N}^{ion}=\cos\left(min\left(\frac{n_{N}^{ion}}{\alpha },\frac{\pi }{2}\right)\right)$$

The final clash-scoring function is:

$$\begin{array}{l} \;f_{clash}^{water}=f_{short}^{water}\\ f_{clash}^{ion}=f_{short}^{ion}\times f_{N}^{ion} \end{array}$$

(5) Combined Score and Classification

For each species (*S*), we compute the combined assignment score:

$$Score^{S}=f_{distance}^{S}\times f_{counts}^{S}\times f_{charge}^{S}\times f_{clash}^{S}$$

The species with the highest *Score*^*S*^ was assigned to the site. To quantify the assignment’s reliability, we compared the top score $\left(score_{Max}^{S}\right)$ to the second highest $\left(score_{2nd}^{S}\right)$. Specifically, we defined the confident score (*CS*) as:

$$CS={log_{2}}^{\left(\frac{score_{Max}^{S}}{score_{2nd}^{S}}\right)}$$

Such that *CS* ≥ 0.6 (a ratio ≥2^0.6^ ≈ 1.5) indicates a high-confidence assignment. All sites with *CS* < 0.6 or those assigned as ions were further examined by manual inspection of coordination geometry and local density (Figure S6D). The expected geometry for water molecules is tetrahedral coordination, whereas for Mg^2+^, K^+^, and Na^+^ ions, we employed the octahedral coordination geometries. For the detailed score and geometry information, please refer to Data S2.

#### Identification of positional overlapped water

To evaluate the spatial position of water molecules across catalytic states of RNA polymerase II, we developed a structural alignment and distance analysis workflow. The procedure comprises the following steps:

Local structural alignment: Given the conformational differences between the pre- and post-catalysis structures, local structural alignment was performed prior to water mapping to enable accurate identification of positionally overlapped water molecules. Specifically, three independent local alignments were conducted, each focusing on a distinct subunit—Rpb1, Rpb2, and Rpb3—resulting in three corresponding pairs of locally aligned structures.

Water Molecule Extraction and Mapping: water molecules were extracted from each pair of structures. Each water molecule in the pre-catalysis structure was then spatially compared against all water molecules in the post-catalysis structure. A water pair was considered positionally overlapped if the distance between their oxygen atoms was below a defined threshold (typically 1.5 Å). In cases where a water molecule from one structure had multiple potential overlapping partners in the other, only the closest pair was retained to avoid redundancy. The results from three local-region alignments were compiled into an Excel table (Data S3), listing individual water molecule identifiers, coordinates, and matched pairs across the two states.

#### MD simulation of Pol II elongation complex

The starting model is based on our high-resolution cryo-EM structure, including resolved water molecules. Short missing loops in Rpb1, Rpb2, and Rpb8 were modeled using ChimeraX^40^ and Modeller.^53^ Due to a large unresolved gap in the cryo-EM structure, Rpb4 was modeled as two separate chains. Missing heavy atoms were added using PDBFixer,^54^ Rpb1 residue H1085 was protonated,^55^ and Zn-coordinated cysteines were deprotonated.

For the MD simulation, the Pol II elongation complex was placed in a triclinic box with a 15-Å space between the complex and the edge of the box and solvated with water molecules. Na^+^ and Cl^–^ ions were added to neutralize the system and achieve a final salt concentration of 0.15 mol/L. All MD simulations were performed using the GROMACS 2022.5^45^ simulation package, with the Amber14SB^56^ protein force field, OL15^57^ force field for DNA and OL3^58^ force field for RNA. ATP parameters were taken from a published force field,^59^ and water was modeled using the TIP3P^60^ model. Long-range electrostatic interactions were treated using the Particle Mesh Ewald (PME)^61^ method, and Van der Waals interactions were computed with a 12-Åcutoff.

The system underwent an initial energy minimization using a steepest descent algorithm for 10,000 steps. The LINCS^62^ algorithm was then applied to constrain bonds involving hydrogen atoms during subsequent steps. A 1-ns NVT MD simulation was performed with position restraints (force constant = 1,000 kJ mol^-1^ nm^-2^) on all heavy atoms as well as the oxygen atoms in the pre-placed water molecules from the cryo-EM structure. This was followed by a 1-ns NPT equilibration under the same position restraint settings. A velocity-rescaling^63^ thermostat (coupling constant = 0.1 ps) was used to maintain a temperature of 300 K, and the Berendsen barostat^64^ was applied during NPT equilibration, with a reference pressure of 1 bar and coupling constant of 0.5 ps. Subsequently, 25 independent production MD simulations were performed under NVT conditions at 300 K, each initiated with different velocities. During these simulations, heavy atoms (excluding water molecules) were restrained with a moderate restraint force constant of 209.2 kJ/mol⋅nm^2^ (0.5 kcal/mol⋅Å^2^), as described in a previous report.^65^ Frames were saved at 5-ps intervals. Temperature annealing from 50 K to 300 K was applied during the first 2.5 ns. The first 10 ns of each production run were discarded, and the following 30 ns were used for the final analysis. To ensure a maximum lag time of 10 ns for MSD calculations, the final 10 ns were excluded as starting points, resulting in an effective simulation length of 20 ns. In total, 500 ns of effective simulation time was accumulated across all production runs.

Mean squared displacements (MSD) were calculated using a hydration site approach. For instance, to analyze the diffusion properties of X water, the hydration site was defined as a sphere with a 1.5 Å radius centered on the X position cryo-EM structure. During simulations, any water molecule entering this hydration site was tracked, and its subsequent displacement was included in the MSD calculation for the X water hydration site. Because positional restraints were applied to heavy atoms, structural alignment was not required. The residence time was defined as the maximum duration for which the MSD remained below 10 Å^2^. The maximum lag time to calculate MSD was 10 ns; any water positions with a residence time exceeding 10 ns were recorded as 10 ns. For bulk water, the MSD exceeded 10 Å^2^ within even the smallest saving interval (5 ps), which is consistent with the previously reported result^66^ showing that the diffusion constant for the TIP3P water in bulk is overestimated at approximately 5×10^5^ cm^2^ s^-1^. To enable comparison, we set up a pure water system within a cubic box measuring 10 nm per side. Using the same MD procedure, except for adjusting the saving interval to 1 ps, we calculated the residence time for bulk water to be 3 ps.

### Quantification and Statistical Analysis

For cryo-EM structure determination, the number of particles contributing to each 3D reconstruction is summarized in Table 1 and detailed in Figures S1, S2, and S9. Resolution estimation was performed using Fourier shell correlation (FSC) analysis in cryo-SPARC,^39^ following standard procedures.

To assess the reliability of candidate densities corresponding to water molecules and ions, signal-to-noise ratios were quantified using Q-scores calculated with the SWIM program^44^ in UCSF Chimera.^41^ All Q-score values are provided in Data S1. For assignment of solvent species, we developed an assignment scoring scheme (see method details) to estimate the likelihood of each water or ion placement; the resulting scores are reported in Data S2.

To further evaluate the conservation and positional consistency of water molecules across different EC structures, we established a structural alignment and distance analysis workflow. Pairwise distance measurements for overlapping water positions are analyzed in Data S3.

## Supplemental Information

Supplemental information can be found online at https://doi.org/10.1016/j.molcel.2026.04.007.

## Figures and Tables

**Table 1:** Cryo-EM data collection, refinement, and validation statistics

Parameters	Pre-catalysis (with Elf1) EC core	Pre-catalysis (with Elf1) nucleic acid	Pre-catalysis (Elf1-free) EC core	Pre-catalysis (Elf1-free) nucleic acid	Pre-catalysis (Elf1-free) Rpb4/7	Pre-catalysis (Elf1-free) Rpb9	Pre-catalysis (Elf1-free) Rpb12/Wall	Pre-catalysis (Elf1-free) Jaw/Rpb9	Pre-catalysis (Elf1-free) composite	Post-catalysis
**PDB**	PDB: 9SV6	N/A	PDB: 9QEB	N/A	N/A	N/A	N/A	N/A	N/A	PDB: 9RYB
**EMDB**	EMDB: 55239	EMDB: 55240	EMDB: 53053	EMDB: 53064	EMDB: 53056	EMDB: 53060	EMDB: 53063	EMDB: 53062	EMDB: 53057	EMDB: 54374
Data collection										
Microscope	Titan Krios	Titan Krios	Titan Krios	Titan Krios	Titan Krios	Titan Krios	Titan Krios	Titan Krios	Titan Krios	Titan Krios
Detector	Falcon 4	Falcon 4	Gatan K3	Gatan K3	Gatan K3	Gatan K3	Gatan K3	Gatan K3	Gatan K3	Gatan K3
Cs (mm)	2.7	2.7	2.7	2.7	2.7	2.7	2.7	2.7	N/A	2.7
Magnification	130,000	130,000	105,000	105,000	105,000	105,000	105,000	105,000	N/A	105,000
Pixel size (A)	0.932	0.932	0.829	0.829	0.829	0.829	0.829	0.829	N/A	0.825
Electron dose (e^−^/ Å^2^)	50	50	50	50	50	50	50	50	N/A	50
Defocus (μm)	−0.5 to 2.5	−0.5 to 2.5	−0.5 to 2.5	−0.5 to 2.5	−0.5 to 2.5	−0.5 to 2.5	−0.5 to 2.5	−0.5 to 2.5	N/A	−0.5 to 2.5
Micrograph	16,482	16,482	10,599	10,599	10,599	10,599	10,599	10,599	N/A	18,913
Reconstruction										
Particles	360,403	67,830	692,163	50,808	107,723	130,110	143,632	134,532	N/A	743,682
Symmetry	C1	C1	C1	C1	C1	C1	C1	C1	N/A	C1
Resolution (Å)	1.96	2.38	2.26	3.12	3.11	3.05	3.03	3.00	N/A	2.33
B-factor (Å^2^)	46.0	40.8	73.6	57.8	86.7	150.0	117.9	100.50	N/A	71.2
Model composition										
Number of atoms	35,033	N/A	33,759	N/A	N/A	N/A	N/A	N/A	N/A	33,090
Protein residues	3,983	N/A	3,908	N/A	N/A	N/A	N/A	N/A	N/A	3,931
Nucleotides	97	N/A	97	N/A	N/A	N/A	N/A	N/A	N/A	54
Ligand	1 (ATP)	N/A	1 (ATP)	N/A	N/A	N/A	N/A	N/A	N/A	1 (PPi)
Bonds RMSD										
Bonds lengths (Å)	0.023	N/A	0.003	N/A	N/A	N/A	N/A	N/A	N/A	0.012
Bonds angles (?)	0.93	N/A	0.628	N/A	N/A	N/A	N/A	N/A	N/A	0.521
Validation										
MolProbity score	1.48	N/A	1.65	N/A	N/A	N/A	N/A	N/A	N/A	1.97
Clash score	6.31	N/A	9	N/A	N/A	N/A	N/A	N/A	N/A	7.28
Rotamer outliers	0.2%	N/A	1.0%	N/A	N/A	N/A	N/A	N/A	N/A	3.7%
C-beta outliers	0.0%	N/A	0.0%	N/A	N/A	N/A	N/A	N/A	N/A	0.0%
Ramachandran plot										
Favored (%)	97.3%	N/A	97.0%	N/A	N/A	N/A	N/A	N/A	N/A	97.0%
Allowed (%)	2.7%	N/A	3.0%	N/A	N/A	N/A	N/A	N/A	N/A	2.9%
Outlier (%)	0.0%	N/A	0.1%	N/A	N/A	N/A	N/A	N/A	N/A	0.1%

## Data Availability

Cryo-EM density maps and corresponding atomic coordinates have been deposited in the Electron Microscopy Data Bank (EMDB) and Protein Data Bank (PDB), respectively. Pre-catalysis (Elf1-bound): 1.96 Å high-resolution map of the RNA Pol II EC in the presence of Elf1 (EMDB: EMD-55239) and corresponding atomic model (PDB: 9SV6). Local refinement map of the nucleic acid scaffold has also been deposited (EMDB: EMD-55240). Pre-catalysis (Elf1-free): 2.26 Åhigh-resolution map of the RNA Pol II EC in the absence of Elf1 (EMDB: EMD-53053) and corresponding atomic model (PDB: 9QEB). A composite map of the global RNA Pol II EC is deposited under accession code EMDB: EMD-53057. Additional maps of flexible regions are available: nucleic acid scaffold (EMDB: EMD-53064), Rpb4/7 (EMDB: EMD-53056), Rpb9 (EMDB: EMD-53060), Rpb12/Wall (EMDB: EMD-53063), and Jaw/Rpb9 (EMDB: EMD-53062). Post-catalysis: 2.33 Å high-resolution map of the RNA Pol II EC (EMDB: EMD-54374) and corresponding atomic model (PDB: 9RYB). This paper does not report original code. Any additional information required to reanalyze the data reported in this paper is available from the lead contact upon request.

## Highlights

- High-resolution cryo-EM structures reveal functional waters in RNA Pol II elongation
- Waters play evolutionarily conserved critical roles in RNA Pol II catalysis
- Waters are crucial for substrate recognition and trigger-loop assembly
- Waters mediate interactions at subunit and protein-nucleic acid interfaces

## In brief

The roles of water molecules in transcription have long been overlooked due to resolution limitations. Li et al. resolve high-resolution cryo-EM structures of RNA polymerase II and visualize previously undetected water molecules. These waters play essential roles in RNA polymerase II catalysis and in mediating interactions within the transcription machinery.
